# HOPE Mitigates Ischemia-Reperfusion Injury in Ex-Situ Split Grafts: A Comparative Study With Living Donation in Pediatric Liver Transplantation

**DOI:** 10.3389/ti.2024.12686

**Published:** 2024-06-07

**Authors:** Guillaume Rossignol, Xavier Muller, Mathias Ruiz, Sophie Collardeau-Frachon, Natacha Boulanger, Celia Depaulis, Teresa Antonini, Remi Dubois, Kayvan Mohkam, Jean-Yves Mabrut

**Affiliations:** ^1^ Department of General Surgery and Liver Transplantation, Croix-Rousse University Hospital, Hospices Civils de Lyon, Lyon, France; ^2^ The Lyon Cancer Research Centre―Lyon Hepatology Institute, INSERM (National Institute of Health and Medical Research) U1052 UMR 5286, Lyon, France; ^3^ ED 340 BMIC (Integrative and Cellular Molecular Biology), Claude Bernard Lyon 1 University, Villeurbanne, France; ^4^ Department of Pediatric Surgery and Liver Transplantation, Femme Mere Enfant University Hospital, Hospices Civils de Lyon, Lyon, France; ^5^ Pediatric Gastroenterology, Hepatology and Nutrition Unit, Femme Mere Enfant University Hospital, Lyon, France; ^6^ Department of Pathology, Hospices Civils de Lyon, Claude Bernard Lyon 1 University, Lyon, France; ^7^ Department of Anesthesiology, Femme Mere Enfant University Hospital, Lyon, France; ^8^ Department of Hepatology, Croix Rousse University Hospital, Lyon, France

**Keywords:** machine perfusion, organ preservation, split liver transplantation, pediatric liver transplantation, ischemia-reperfusion injury

## Abstract

Optimizing graft preservation is key for ex-situ split grafts in pediatric liver transplantation (PSLT). Hypothermic Oxygenated Perfusion (HOPE) improves ischemia-reperfusion injury (IRI) and post-operative outcomes in adult LT. This study compares the use of HOPE in ex-situ partial grafts to static cold storage ex-situ partial grafts (SCS-Split) and to the gold standard living donor liver transplantation (LDLT). All consecutive HOPE-Split, SCS-Split and LDLT performed between 2018–2023 for pediatric recipients were included. Post‐reperfusion syndrome (PRS, drop ≥30% in systolic arterial pressure) and reperfusion biopsies served as early indicators of IRI. We included 47 pediatric recipients (15 HOPE-Split, 17 SCS-Split, and 15 LDLT). In comparison to SCS-Split, HOPE-Split had a significantly shorter cold ischemia time (CIT) (470min vs. 538 min; *p* =0.02), lower PRS rates (13.3% vs. 47.1%; *p* = 0.04) and a lower IRI score (3 vs. 4; *p* = 0.03). The overall IRI score (3 vs. 3; *p* = 0.28) and PRS (13.3% vs. 13.3%; *p* = 1) after HOPE-Split were comparable to LDLT, despite a longer CIT (470 min vs. 117 min; *p* < 0.001). Surgical complications, one-year graft, and recipient survival did not differ among the groups. In conclusion, HOPE-Split mitigates early IRI in pediatric recipients in comparison to SCS-Split, approaching the gold standard of LDLT.

## Graphical Abstract

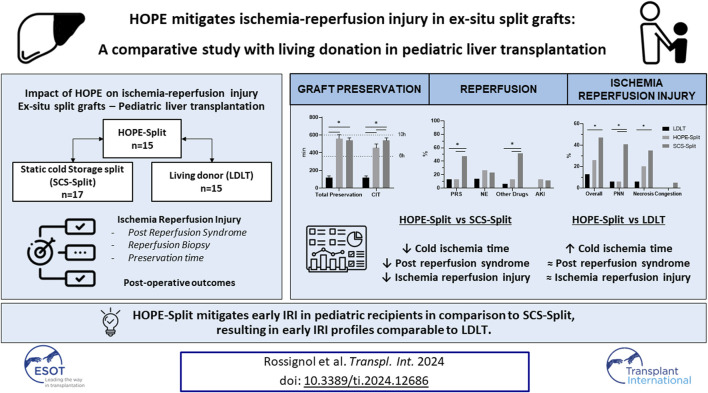

## Introduction

Living donor liver transplantation (LDLT) provides the best achievable outcomes for pediatric recipients [1, 2]. In addition to optimal donor selection, LDLT grafts have a short static cold ischemia time (CIT) resulting in less ischemia-reperfusion injury (IRI) and improved post-LT outcomes [3, 4]. Nevertheless, in France, LDLT accounts for only 12% of all pediatric liver transplantations (PLT) and the majority of PLT are performed with ex-situ split grafts from deceased donors (PSLT) [5]. Pediatric prioritization and strict donor selection have enabled PSLT from deceased donors to yield excellent outcomes although they have not yet reached the benchmarks set by living donation in terms of graft and patient survival. One of the main independent risk factors for early graft loss in PSLT is CIT [6, 7]. One strategy to improve preservation is the use of hypothermic oxygenated perfusion (HOPE), especially in case of ex-situ split procedures. As shown in adult LT, application of HOPE is associated with reduced rates of post-reperfusion syndrome (PRS) [8], histological IRI [9] and improved post-LT outcomes [8, 10, 11]. PRS is also a major determinant of graft survival in PLT with a reported incidence up to 34%. Therefore, PLT may benefit from the implementation of HOPE to mitigate PRS an IRI [12–14]. While the safety and feasibility of HOPE in PSLT have already been established, there is currently no data on the impact of HOPE on early IRI indicators available [9, 15, 16]. Thus, this study will focus on the impact of HOPE on IRI in ex-situ split grafts for pediatric recipients in comparison to the gold standard LDLT.

## Method

### Study Design

This retrospective study focuses on PSLT and aims at investigating the impact of HOPE on ex-situ split grafts from deceased donors (HOPE-Split) in comparison to the gold standard LDLT and ex-situ grafts splitted during SCS (SCS-Split).

We included all PSLT performed prospectively from 2018 to 2023 with at least 6 months follow-up, including LDLT, SCS-Split and HOPE-Split procedures (Supplementary Figure S1). Of note, 5 *in-situ* splits were performed at our center during the study period and were excluded due to small sample size.

Graft selection for deceased donor was based on current data [17] relying on donor age (<45 years), body mass index (<25 kg/m^2^), intensive care unit stay (<7 days), cardiac arrest and donor biology.

The implementation of HOPE in the pediatric setting followed the IDEAL recommendations for surgical innovation [18]. The safety and benefit of HOPE has been established in adult LT [11, 19] allowing for its application in pediatric LT. The safety of HOPE-Split has been previously assessed in case series and the surgical technique has been refined through Stage I and IIa studies [9, 16]. To further investigate this strategy and expand its indications (Stage IIb), this study focused on SPLT, aiming to compared HOPE-Split to the gold standard LDLT, and was approved by the local ethics committee (CSEHCL_21_202).

### HOPE Split Procedure

The procedure for liver graft splitting during HOPE has been previously standardized and reported [16]. The first step of the Split procedure was performed during static cold storage. It consisted in the pedicular dissection aiming at identifying the portal vein and the hepatic artery division. The portal vein was not divided allowing for the perfusion of both partial grafts with a single cannula. A cholangiography was performed to assess biliary anatomy prior to parenchymal transection. The second step, namely parenchymal transection, was performed during HOPE. Both grafts were perfused at a pressure of a maximum of 5 mmHg with a portal flow ranging from 200 to 300 mL/min.

Since 2022, in line with the findings from *Ravaioli et al.* in adult LT [8], HOPE was initiated at the beginning of the back table preparation [20].

### Endpoints

We specifically investigate the impact of HOPE on surrogate markers of early IRI in pediatric recipients, namely post-reperfusion syndrome (PRS) and histological ischemia-reperfusion injuries. PRS in pediatric recipients was defined according to *Zhang et al.* as a drop of systolic arterial pressure (SAP) of more than 30% within the first 5 min following reperfusion [13]. To refine PRS assessment, increase of norepinephrine (NE), the use of other vasoactive drugs such as adrenaline, median post-reperfusion SAP or mean arterial pressure (MAP) and acute kidney injury requiring dialysis (AKI) were also evaluated. IRI based on reperfusion biopsy were assessed as previously described [9]. A blinded reading by one experienced pathologist was performed and histological IRI was ranked as grade 0 for absence of IRI, grade 1 for minimal IRI, grade 2 for mild IRI, grade 3 for moderate IRI and grade 4 for severe IRI. A histological IRI ≥ grade 3 (moderate to severe) was considered as a high-grade injury. Overall IRI score based on each compartment evaluation (Neutrophilic infiltrate, necrosis, congestion) was calculated.

To assess the impact of HOPE on graft preservation we evaluated CIT and total preservation time. CIT was defined by static cold storage duration from *in situ* cold flush in the donor to either the beginning of HOPE or the implantation of the graft.

In addition, early graft function was assessed and graded according to the Olthoff criteria (Early Allograft Dysfunction [EAD]) [21] and to the LGraft7 score [22]. 1 year graft and patient survival were assessed as well as overall morbidity using the Comprehensive Complication Index (CCI^©^) [23] and the Clavien-Dindo classification [24].

### Statistical Analysis

Categorical variables were expressed in quantities and percentages while continuous variables were expressed as median with interquartile range (IQR). Continuous variables were compared using the Kruskal-Wallis with *post hoc* Dunn’s test to compare the 3 study groups or with the Mann Whitney test. Categorical variables were compared using the chi-square test or the Fisher’s exact test. Kaplan Meier curves with a log rank test were used to compare graft and patient survival.

*p*-values <0.05 were considered statistically significant. Statistical analysis was performed using IBM SPSS Statistics for Windows (Version 26.0. Armonk, NY: IBM Corp) and GraphPad Prism (Version 8.0.0 for Windows, GraphPad Software, San Diego, California United States).

## Results

### Study Cohort

Donor characteristics were similar between the HOPE-Split and SCS-Split group (Table 1), with a median age of 20 years, a median BMI of 21.6 kg/m^2^. The main donor cause of death was traumatic (59%), and 18.7% of donors had a cardiac arrest prior to graft procurement.

**TABLE 1:** Donor-recipients characteristics, surgical data and post-operative outcomes.

	LDLT *n* = 15	HOPE-Split *n* = 15	SCS-Split *n* = 17	HOPE-Split vs SCS-Split p value	HOPE-Split vs LDLT p value
Donor Characteristics					
Age (y)	36 [30–38]	21 [17–28]	20 [18–30]	0.91	<0.001
Sex (M)	66.7 (10)	73.3 (11)	41.2 (7)	0.07	0.69
BMI (kg/m^2^)	23.1 [20.4–26]	18.9 [17.4–23.5]	22.5 [20.1–22.9]	0.71	0.10
COD					
Traumatic	-	66.7 (10)	52.9 (9)	0.43	-
Hypoxic Brain Injury	-	6.7 (1)	29.4 (5)	0.18	-
CerebroVascular	-	26.7 (4)	17.6 (3)	0.54	-
Cardiac Arrest	-	13.3 (2)	23.5 (4)	0.46	-
Recipient Characteristics					
Age (months)	17 [9.5–56.5]	43 [19.5–51]	21 [13–38]	0.17	0.39
Sex (M)	33.3 (5)	26.7 (4)	41.2 (7)	0.38	0.69
Weight (kg)	10.5 [7.5–16]	15 [10–17]	10 [8.5–14]	0.14	0.46
PELD	16 [9–21]	19 [16–21]	23 [15–29]	0.35	0.15
BA	60 (9)	57.1 (8)	47 (8)	0.46	0.71
Tumor	20 (3)	0 (0)	5.8 (1)	0.34	0.68
Urgency	20 (3)	46.7 (7)	58.8 (10)	0.49	0.12
ALF	0 (0)	14.2 (2)	17.6 (3)	0.74	0.14
reLT	6.7 (1)	28.5 (4)	0 (0)	0.02	0.14
Liver Transplantation					
Preservation Time (min)	117 [99–139]	568 [525–608]	538 [514–567]	0.19	<0.001
CIT (min)	117 [99–139]	470 [376–505]	538 [514–567]	<0.001	<0.001
WIT (min)	32 [29–36]	36 [34–39]	33 [31–37]	0.04	0.03
Transfusion (mL/kg)	24 [18–37]	35 [23–47]	47 [27–69]	0.24	0.17
GRWR (%)	2.2 [1.5–3.2]	2.4 [1.8–2.6]	2.8 [2.4–3.2]	0.11	0.95
Post-operative Outcomes					
EAD	20 (3)	66.7 (10)	70.6 (12)	0.81	0.01
LGraft7^a^	−3.31 [-3.69;-2.02]	−3.05 [-4.17;-2.25]	−3.25 [-4.26;-2.13]	1	0.74
Risk LGraft7^b^	3.5 [2.4–11.7]	6.6 [1.5–10.3]	3.7 [1.4–10.6]	0.78	0.98
PNF	0 (0)	6.6 (1)	5.8 (1)	0.93	0.31
PRS	13.3 (2)	13.3 (2)	47 (8)	0.04	1
AKI	0 (0)	13.3 (2)	11.8 (2)	0.89	0.14
Early Laparotomy	46.7 (7)	40 (6)	35.3 (6)	0.78	0.71
Biliary Complications	40 (6)	33.3 (5)	35.3 (6)	0.91	0.71
Anastomotic stricture	40 (6)	20 (3)	17.6 (3)	0.86	0.23
Non anastomotic stricture	0 (0)	13.3 (2)	17.6 (3)	0.73	0.14
Arterial Complications	0 (0)	20 (3)	11.8 (2)	0.52	0.07
Stenosis	0 (0)	13.3 (2)	0 (0)	0.12	0.14
Thrombosis	0 (0)	0 (0)	11.8 (2)	0.17	-
Pseudoaneurysm	0 (0)	6.7 (1)	0 (0)	0.28	0.31
CCI 3 months	53 [39–79]	68 [41–99]	71 [46–90]	0.71	0.37
CCI 12 months	71 [44–86]	94 [44–99]	92 [63–99]	1	0.27
Graft Survival (3 m)	100 (15)	86.7 (13)	94.1 (16)	0.47	0.14
Patient survival (3 m)	100 (15)	86.7 (13)	94.1 (16)	0.47	0.14
Graft survival (1 year)	100 (15)	86.7 (13)	88.2 (15)	0.89	0.14
Patient survival (1 year)	100 (15)	86.7 (13)	94.1 (16)	0.47	0.14

Values are expressed as % (n) or median [interquartile range].

(BMI: body mass index, COD: cause of death, BA: biliary atresia; ALF: acute liver failure, reLT: retransplantation, PELD: Pediatric End-Stage Liver Disease, CIT: cold ischemia time, WIT: warm ischemia time, GRWR: graft over recipient weight ratio, EAD: early allograft dysfunction, PNF: primary non function, PRS: post reperfusion syndrome, AKI: acute kindey injury requiring dialysis, CCI: Comprehensive Complication Index).

^a^ Continuous variables were compared using the Mann Whitney test. Categorical variables were compared using the Fisher’s exact test.

^b^ LGRAFT, score was presented (negative value) as well as the risk of graft loss (percentage).

Living donors were mostly the father of the recipient (66.7%), with a median age of 36 years and a median BMI of 23.1 kg/m^2^.

As shown in Table 1, recipient characteristics regarding median age, weight and PELD (Pediatric End Stage Liver Disease) were comparable between groups. The main cause for PSLT was biliary atresia (53%) with significantly more retransplantations in the HOPE-Split group (28.5%; *p* = 0.03). Recipients in both HOPE-Split and SCS-Split presented with a trend toward higher rate of acute liver failure (14.2% and 17.6% vs. 0% respectively; *p* = 0.24) and high urgency listing (46.7% and 58.8% vs. 20% respectively; *p* = 0.08) compared to LDLT recipients.

### Ischemia Reperfusion Injury

#### Graft Preservation

In the HOPE-Split group, HOPE was performed for a median time of 100 min with a significant shorter CIT in comparison to SCS-Split (470 min vs. 538 min; *p* = 0.01). Total ex-vivo preservation time was not significantly different between the two groups (568 min vs. 538 min; *p* = 0.36).

**FIGURE 1:**
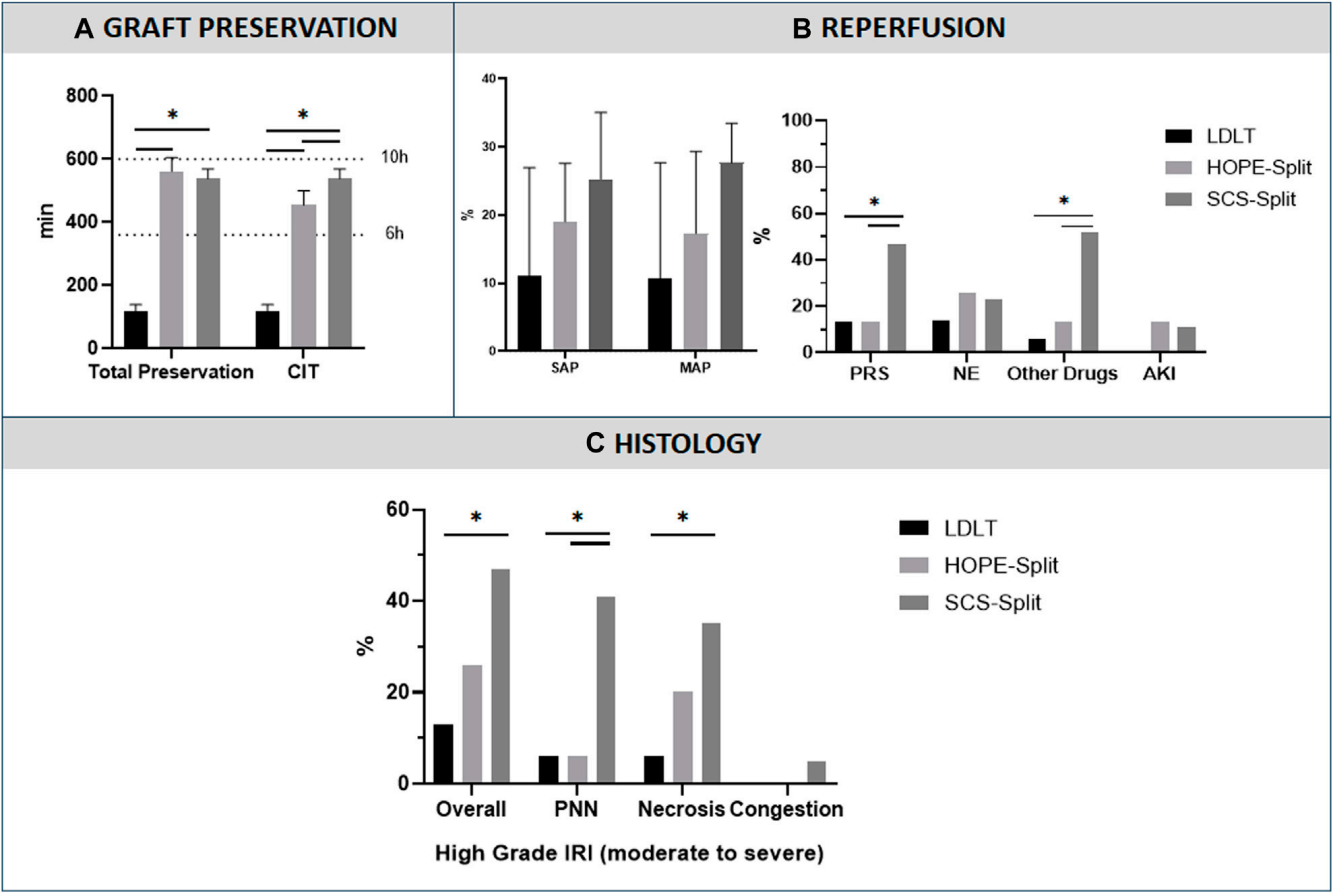
Preservation characteristics and Ischemia-Reperfusion Injury, **(A)** Graft preservation time, **(B)** Post Reperfusion Syndrome, **(C)** Histological analysis of ischemia reperfusion injury. Data are presented as median with interquartile range. (Ex-Vivo: Total Ex-vivo preservation time, CIT: Cold Ischemia time, SAP: Systolic Arterial Pressure, MAP: Mean Arterial Pressure, PRS: Post-reperfusion syndrome, NE: NorEpinephrine; AKI: Acute Kidney Injury requiring dialysis, PNN, Neutrophilic infiltrate, *: p < 0.05)

Overall, the LDLT group presented with the shortest CIT compared to both HOPE-Split and SCS-Split groups (117 min; *p* < 0.001) (Figure 1).

#### Post-Reperfusion Syndrome

The HOPE-Split group showed a significant reduction of PRS compared to the SCS-Split group (13.3% vs. 47.1%; *p* = 0.04) with significantly less additional post-reperfusion vasoactive drugs (13.3% vs. 52.9%; *p* = 0.02) (Figure 1). No difference was observed regarding post-LT AKI (13.3% vs. 11.8%; *p* = 0.89).

In comparison to LDLT, the PRS rate (13.3% vs. 13.3%; *p* = 1), NE increase (26.7% vs. 14.3%; *p* = 0.41) and the use of other vasoactive drugs (13.3% vs. 6.7%; *p* = 0.54) were not significantly different in the HOPE-Split group.

#### Reperfusion Biopsy

The HOPE-Split group exhibited a trend toward less high-grade IRI (moderate to severe, grade ≥3; 26.7% vs. 47.1%; *p* = 0.23) and a significantly lower neutrophilic infiltrate (6.7% vs. 41.2%; *p* = 0.02) with a significantly lower overall IRI score (3 [2–5] vs. 4 [4–7]; *p* = 0.03) compared to SCS-Split (Figure 1).

In comparison to LDLT, HOPE-Split exhibited a trend toward more histological high-grade IRI (26.7% vs. 13.3%; *p* = 0.36) without significant difference regarding the overall injury score (3 [2–5] vs. 3 [2-3]; *p* = 0.28).

### Early Post-Operative Outcomes

**FIGURE 2:**
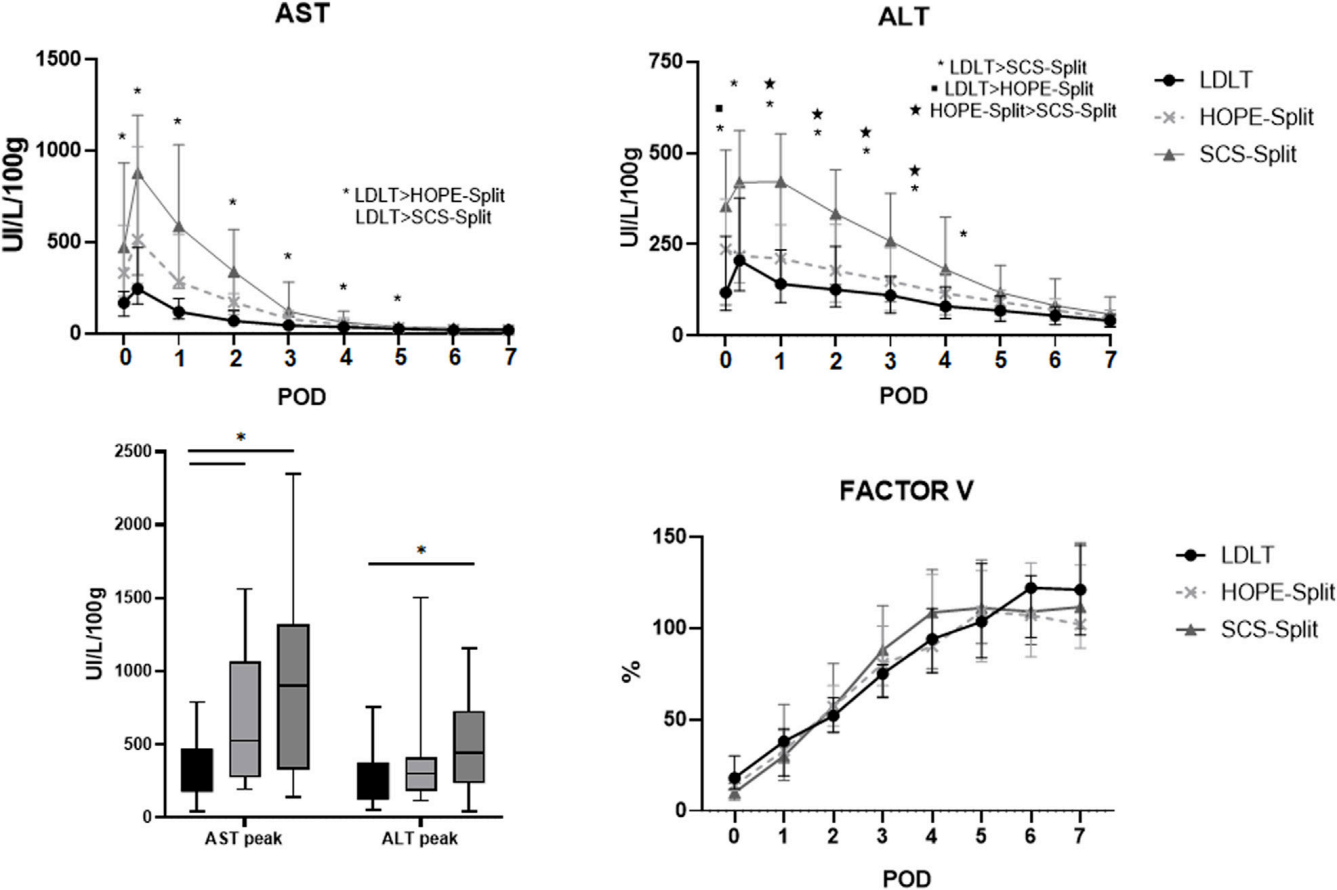
Serum aspartate aminotransferase (AST), alanine aminotransferase (ALT) and Factor V during the first post-operative days. Data are expressed as median and interquartile range. POD, Post-operative days; AST, aspartate aminotransferase; ALT, alanine aminotransferase.

The HOPE-Split group exhibited significantly less ALT release during the first four post-operative days (Figure 2) with a trend toward lower AST and ALT peak (523 UI/L/100 g vs. 909 UI/L/100 g; *p* = 0.30 and 303 UI/L/100 g vs. 440 UI/L/100 g; *p* = 0.19) compared to SCS-Split.

In comparison to LDLT, the HOPE-Split group exhibited a significant higher AST peak (523 UI/L/100 g vs. 244 UI/L/100 g; *p* = 0.01) and ALT peak (303 UI/L/100 g vs. 205 UI/L/100 g; *p* = 0.25) resulting in a significant higher rate of EAD (66.7% vs. 20%; *p* = 0.007).

Factor V normalization was similar between HOPE, SCS and LD (Figure 2).

The HOPE-Split group exhibited similar rates of early laparotomy (40%; *p* = 0.81), biliary complications (33.3%; *p* = 0.92) and arterial complications (20%; *p* = 0.21) compared to both LDLT and SCS-Split (Table 1).

One year graft and patient survival were 86.7% (*n* = 13/15) in the HOPE-Split group without statistically significant differences compared to both LDLT and SCS-Split (Table 1; Supplementary Figure S1).

## Discussion

This is the first study to investigate the impact of HOPE on early IRI in PSLT by a direct comparison with the gold standard LDLT and standard ex-situ split grafts. We were able to show that HOPE-Split significantly reduced PRS and IRI on reperfusion biopsy in comparison to SCS-Split, resulting in comparable IRI profiles to LDLT.

Graft preservation remains a key challenge in PSLT as CIT has been shown to be an independent risk factor for graft loss [6]. In addition, *Lauterio et al.* [7] recently showed that CIT >6 h and >10 h were associated with graft failure in a cohort of in-situ PSLT. Besides, CIT is related to PRS [12] and IRI which are known risk factors for graft loss [13]. HOPE has been shown to improve graft preservation by actively oxygenating the graft associated with shorter CIT [8], translating into decreased PRS [10, 25, 26], decreased EAD, ischemic type biliary complications and graft loss [11] in adult LT. In our institution, we therefore implemented HOPE for ex-situ split liver grafts since 2020 aiming at improving graft preservation in PSLT. In the present study we compared for the first time HOPE-Split to LDLT gold standard and to SCS-Split to evaluate the impact of HOPE on early ischemia-reperfusion events, namely, PRS and IRI injury on reperfusion biopsy.

First, the HOPE-Split group exhibited lower rates of PRS, as well as improved hemodynamic stability upon reperfusion compared to SCS-Split. This observation is in line with previous data from adult split transplantation using HOPE [10, 26]. In addition, we observed a lower grade of histological IRI and less neutrophilic infiltrate in the HOPE-Split group. This allowed the HOPE-Split grafts to approach outcomes with LDLT regarding early IRI without statistically significant differences in PRS and IRI on reperfusion biopsy. These clinical observations are supported by experimental data showing a reduction of mitochondrial damage with HOPE which translates into a reduction of the hepatic inflammasome [27, 28]. Indeed, HOPE replaces cold ischemia by an active oxygenation of the graft during preservation thus improving mitochondrial function, uploading the ATP cellular pool [28, 29] and mitigating IRI [10, 25, 26]. Applying HOPE during ex-situ liver splitting thus combines the benefit of shorter CIT, inherent to this strategy, to mitochondrial metabolism recovery.

Second, all PSLT groups showed a 1-year graft survival rate of >85% which is comparable to the data from the ELTR registry and the UNOS data base [3, 4]. Improved preservation characteristics did not result in a decrease in overall morbidity or mortality in our study. Additionally, meaningful statistical adjustments for recipient risk factors were not possible due to the small sample size. Nevertheless, early IRI events such as PRS [12, 13] and IRI on reperfusion biopsy [30] have been shown to significantly impact long-term post-LT outcomes in larger cohorts, including LDLT [12] and serve as early surrogates of graft quality.

Altogether, these data suggest that HOPE-Split could mitigate left partial liver graft IRI similar to the impact of HOPE in whole liver transplantation [8, 31]. The presented results demonstrate that HOPE, by replacing CIT during ex-situ liver splitting, may be a promising strategy to expand donor selection criteria especially for split liver grafts [14]. Besides, performing back-table preparation during active perfusion can further improve graft preservation allowing for a CIT <6 h, similar to in-situ split grafts [8, 20], which may facilitate logistics. Graft evaluation [32] and specific scenarios that might benefit the most from HOPE still need to be explored to safely increase the donor pool for pediatric recipients through tailored preservation strategies [14]. In addition to PLT, HOPE may also facilitate the access to partial grafts for adult recipients with oncological indications in the context of the RAPID procedure (Resection And Partial Liver Segment 2/3 Transplantation with Delayed Total Hepatectomy) [33].

Our study has some limitations inherent to its retrospective design. A small sample size and a focus on short-term follow-up do not allow to draw robust conclusion regarding the potential benefit of HOPE on long-term clinical outcomes. According to the IDEAL framework for surgical innovation [18], larger scale prospective trials (Stage III) are mandatory to provide robust data on the independent effect of HOPE in PLT. This will soon be assessed in a multicenter national prospective randomized trial (HOPE-Split) supported from the French Ministry of Health through a grant from the National Hospital Clinical Research Program. Regarding PRS, there exist several definitions in the literature and preoperative management may differ from center to center [13]. However, in this single center study, there was a protocolized standard of care for PRS management in all recipients included.

In conclusion, HOPE-Split allows to reduce PRS rates and histological IRI in comparison to SCS-Split, resulting in early IRI profiles comparable to LDLT. Improving early IRI with HOPE in PSLT could benefit high-risk donor-recipient scenarios and allow expanding selection criteria for ex-situ split grafts. Future multicenter trials should now evaluate long-term outcomes of HOPE-Split in larger cohorts and identify specific situation that might benefit the most from dynamic preservation.

## Data Availability

The raw data supporting the conclusions of this article are not publicly available but will be made available by the corresponding author upon reasonable request.

## Supplementary Material

SUPPLEMENTARY FIGURE S1 Study flow chart.

SUPPLEMENTARY FIGURE S2 One year graft and patient survival.

## Abbreviations

CCI, comprehensive complication index; CIT, cold ischemia time; EAD, early allograft dysfunction; HOPE, hypothermic oxygenated perfusion; IQR, inter quartile range; IRI, ischemia reperfusion injury; LDLT, living donor liver transplantation; LT, liver transplantation; MAP, mean arterial blood pressure; NE, NorEphinephrine; PELD, pediatric end-stage liver disease; PNF, primary non function; PRS, post reperfusion syndrome; PSLT, pediatric split liver transplantation; SAP, systolic arterial blood pressure; SCS, static cold storage.
