# Supplementary figures and images for: HOPE Mitigates Ischemia-Reperfusion Injury in Ex-Situ Split Grafts: A Comparative Study With Living Donation in Pediatric Liver Transplantation
Source: Transpl Int. 2024 Jun 7;37:12686. doi: 10.3389/ti.2024.12686 (PMC11190067; doi:10.3389/ti.2024.12686)

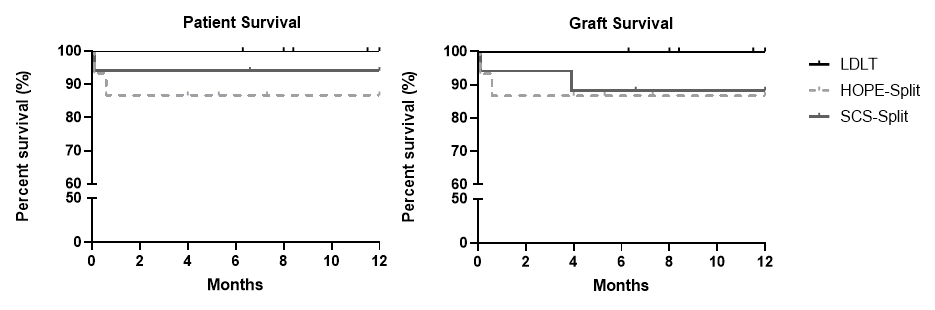

Supplement: Supplementary file 1 [file Image2.TIF]

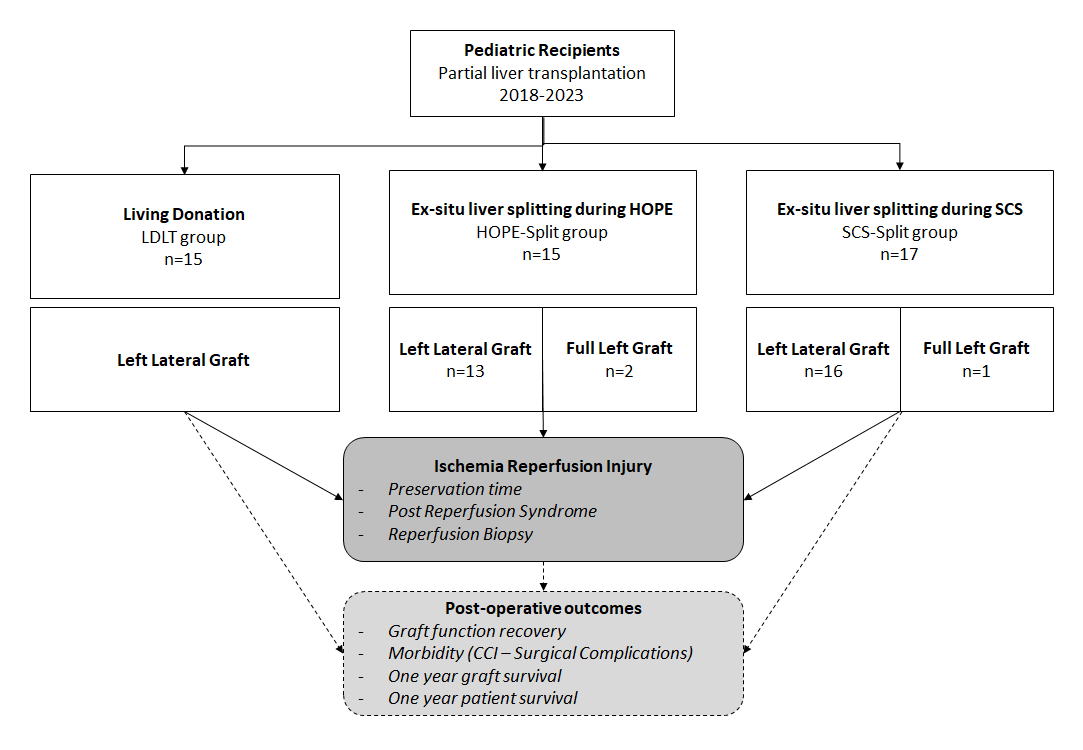

Supplement: Supplementary file 2 [file Image1.TIF]
